# Psilocybin therapy increases cognitive and neural flexibility in patients with major depressive disorder

**DOI:** 10.1038/s41398-021-01706-y

**Published:** 2021-11-08

**Authors:** Manoj K. Doss, Michal Považan, Monica D. Rosenberg, Nathan D. Sepeda, Alan K. Davis, Patrick H. Finan, Gwenn S. Smith, James J. Pekar, Peter B. Barker, Roland R. Griffiths, Frederick S. Barrett

**Affiliations:** 1 Department of Psychiatry and Behavioral Sciences, Johns Hopkins University School of Medicine, Baltimore, USA; 2 Center for Psychedelic & Consciousness Research, Johns Hopkins University School of Medicine, Baltimore, USA; 3 Department of Radiology and Radiological Science, Johns Hopkins University School of Medicine, Baltimore, USA; 4 Department of Psychology, University of Chicago, Chicago, USA; 5 College of Social Work, The Ohio State University, Columbus, USA; 6 F.M. Kirby Research Center for Functional Brain Imaging, Kennedy Krieger Institute, Baltimore, USA; 7 Department of Neuroscience, Johns Hopkins University School of Medicine, Baltimore, USA

**Keywords:** Neuroscience, Predictive markers

## Abstract

Psilocybin has shown promise for the treatment of mood disorders, which are often accompanied by cognitive dysfunction including cognitive rigidity. Recent studies have proposed neuropsychoplastogenic effects as mechanisms underlying the enduring therapeutic effects of psilocybin. In an open-label study of 24 patients with major depressive disorder, we tested the enduring effects of psilocybin therapy on cognitive flexibility (perseverative errors on a set-shifting task), neural flexibility (dynamics of functional connectivity or dFC via functional magnetic resonance imaging), and neurometabolite concentrations (via magnetic resonance spectroscopy) in brain regions supporting cognitive flexibility and implicated in acute psilocybin effects (e.g., the anterior cingulate cortex, or ACC). Psilocybin therapy increased cognitive flexibility for at least 4 weeks post-treatment, though these improvements were not correlated with the previously reported antidepressant effects. One week after psilocybin therapy, glutamate and *N*-acetylaspartate concentrations were decreased in the ACC, and dFC was increased between the ACC and the posterior cingulate cortex (PCC). Surprisingly, greater increases in dFC between the ACC and PCC were associated with less improvement in cognitive flexibility after psilocybin therapy. Connectome-based predictive modeling demonstrated that baseline dFC emanating from the ACC predicted improvements in cognitive flexibility. In these models, greater baseline dFC was associated with better baseline cognitive flexibility but less improvement in cognitive flexibility. These findings suggest a nuanced relationship between cognitive and neural flexibility. Whereas some enduring increases in neural dynamics may allow for shifting out of a maladaptively rigid state, larger persisting increases in neural dynamics may be of less benefit to psilocybin therapy.

## Introduction

Recently, classic psychedelics (serotonin 2A or 5-HT_2A_ agonists such as psilocybin, lysergic acid diethylamide or LSD, and *N*,*N*-dimethyltryptamine or DMT) have shown potential efficacy for treating a variety of psychiatric disorders [1–6], including major depressive disorder (MDD). Whereas typical antidepressant treatments such as selective serotonin reuptake inhibitors (SSRIs) can take weeks to months to take effect, two administrations of psilocybin coupled with extensive psychotherapy appear to have rapid and enduring antidepressant effects (e.g., lasting weeks to months after the acute effects [2, 3] and at least as effective as a SSRI [7]). Despite these striking effects of psychedelic therapy, the cognitive and neural mechanisms underlying their enduring efficacy are poorly understood.

A potential transdiagnostic neuropsychological mechanism that may be targeted by psychedelic therapy is cognitive flexibility. Cognitive flexibility is broadly defined as the ability to adaptively switch between different cognitive operations in response to changing environmental demands, and it is typically measured as perseveration on prior rules in set shifting or rule switching tasks (for review, see [8]). Impairments in cognitive flexibility have been found in depression [9, 10], as well as obsessive-compulsive disorder [11] and substance use disorders [12], which are disorders that may also be amenable to psychedelic therapy [1, 5, 6]. Cognitive flexibility deficits may precede the onset of other symptoms in depression [13], have been identified across the lifespan in patients with depression [14, 15], and may represent an endophenotype for depression [16]. Although cognitive flexibility may not always be directly related to global measures of symptomology for a given disorder [17], the capacity to readily shift between different mental states could especially be useful in the context of psychotherapy [18]. Furthermore, the related concept of psychological flexibility has been found to mediate the relationship between acute psychedelic effects and improvements in depression and anxiety [19]. Antidepressant treatments including ketamine [20], SSRIs [21–23], duloxetine (a serotonin and norepinephrine reuptake inhibitor) [17], and repetitive transcranial magnetic stimulation [22] have been found to improve cognitive flexibility, perhaps by driving plasticity of the hippocampus [24, 25], a structure implicated in depression [26] and flexible behavior [27]. Nevertheless, many patients remain treatment-refractory for cognitive deficits [28, 29], necessitating research on novel treatments.

Although it is unclear what the enduring effects of psychedelic therapy are on cognitive flexibility in humans, the acute effects of 5-HT_2A_ receptor modulation on cognitive flexibility are mixed. Blockade of 5-HT_2A_ receptors has been found to both impair [30, 31] and enhance cognitive flexibility in animal models [32–34]. In contrast, activation of 5-HT_2A_ receptors with psychedelic drugs has been found to acutely impair cognitive flexibility in humans [35] and impair or have no impact on cognitive flexibility in animals [36, 37]. Moreover, psychedelics also activate the 5-HT_2C_ receptor, and the relationship between modulation of this receptor and cognitive flexibility is complex. In animal models, 5-HT_2C_ blockade alone has been found to enhance [30, 38] or have no impact cognitive flexibility [34]. However, one study found that 5-HT_2C_ blockade during co-administration of a psychedelic impaired cognitive flexibility [36] when the administration of the psychedelic alone had no impact, suggesting that coactivation of the 5-HT_2C_ receptor may be protective against impairments in cognitive flexibility from 5-HT_2A_ activation. Together, these findings suggest that acute activation or blockade of receptors targeted by psychedelics can bidirectionally modulate cognitive flexibility, though it remains unclear whether any of these effects extend into the days following psychedelic administration.

Activity of the anterior cingulate cortex (ACC) and its interactions with other regions [39, 40] are known to support cognitive flexibility, and single units of the ACC are involved in search for new rules to promote adaptive behavior [41]. Several pieces of evidence suggest that psychedelics modulate the activity of the ACC. In humans, psychedelics have been found to acutely increase ACC activity [42–44] and task-free static functional connectivity (sFC; the temporal coupling between the activity of two regions) between the default mode and salience networks [45–47], the ACC being a major hub of the latter. Increased sFC between the default mode and salience networks [48] and between the ACC and posterior cingulate cortex (PCC; [49]) has also been observed 1 day after DMT administration (in the orally active form, ayahuasca). Like the ACC, activity and sFC of the PCC are consistently altered during acute effects of classic psychedelics [46, 50–52], and concentrations of glutamate and *N*-acetylaspartate (NAA) in the PCC were reduced 1 day post-DMT administration [49]. Finally, sFC between the PCC and the subgenual cingulate, a region ventral to the ACC, was increased 1 day after psilocybin therapy in patients with MDD [53]. The subgenual cingulate is a major target in the treatment of depression [54], and reduced sFC between the subgenual cingulate and ACC has been observed in MDD and is associated with emotional rigidity [55].

Recently, several studies have suggested that psychedelics induce neural plasticity [56–60] (for reviews, see [61, 62]). With markers of neural plasticity difficult to assess in humans, a potential proxy for ongoing shifts in synaptic weighting within and across brain regions may come from measures of neural flexibility in functional magnetic resonance imaging (fMRI) data (i.e., signal variability over time). Different measures of neural flexibility, especially those involving the salience network [63], have been associated with cognitive flexibility [8, 64], and neural flexibility of the ACC, as well as the PCC, has been found to be decreased in MDD [65, 66]. Moreover, psychedelics and other hallucinogens have been found to acutely increase various measures of variance and entropy in brain activity, especially in regions of the salience network including the ACC ([67–71]; though decreases have also been reported [46, 71, 72]). Although the durability of these effects is unclear in patients with MDD, one small study of 12 healthy adults did not find enduring modulation of neural flexibility or ACC function 1 week and 4 weeks after a single dose of psilocybin [73].

Here, we utilized both hypothesis-driven and data-driven approaches of multi-modal brain imaging measurements to examine the enduring effects of psilocybin therapy on cognitive and neural flexibility in patients with MDD. Specifically, we examined the relationship between changes in depression, cognitive flexibility, and both sFC and the dynamics of functional connectivity (dFC; variance in the timeseries of moment-to-moment sFC as measured with fMRI) following psilocybin therapy. In addition, we collected magnetic resonance spectroscopy (MRS) data to characterize psilocybin-induced changes in neurometabolite concentrations in the ACC and hippocampus, regions involved in cognitive flexibility [24, 25, 27, 39–41] with abnormal glutamate and NAA concentrations in depression that are responsive to antidepressant treatments [74–76]. As the MRI was performed at 7 T, there was dropout in the fMRI signal in some parts of the brain including the hippocampus. Thus, we narrowed our focus of sFC and dFC to interactions involving the ACC and PCC.

## Methods

### Participants

Detailed participant information was previously published [3]. Twenty-four participants (eight males) aged 24–59 years (*M* = 39.83, SD = 12.23) with MDD (≥17 on the GRID-Hamilton Depression Rating Scale or GRID-HAMD) completed neuroimaging and/or cognitive tasks (see specific measures for missing data) in an open-label clinical trial (ClinicalTrials.gov Identifier: NCT03181529). This sample size was chosen based on previous work that found a large effect size on the GRID-HAMD [4] (i.e., the primary outcome of this clinical trial). Participants were recruited through advertisements and word-of-mouth referrals. Screening included internet surveys, phone interviews, and in-person medical and psychiatric assessments. Exclusion criteria included current antidepressant medication, substantial lifetime use (>10 total) or recent use (past 6 months) of ketamine or classic psychedelics, a current significant medical condition, personal or family history (first or second degree) of psychotic or bipolar disorders, moderate or severe alcohol or other drug use disorder (including nicotine) in the past year, standard fMRI contraindications (e.g., left-handed, incompatible medical devices), and for women, being pregnant or nursing. This study was approved by the Johns Hopkins Medicine Institutional Review Board, and all participants provided informed consent.

### Psilocybin therapy

Detailed information regarding the full treatment procedure can be found elsewhere [3]. Thirteen and 11 participants were randomly assigned (via urn randomization) to an immediate treatment and delayed treatment group, respectively. After screening, participants in the immediate group completed baseline clinical, cognitive, and neuroimaging measurements followed by ~8 h of preparatory therapy sessions conducted over 2 weeks to build rapport with research personnel. Following these preparatory sessions, participants attended two psilocybin sessions separated by ~1.6 weeks. A moderately high dose (20 mg/70 kg) and a high (30 mg/70 kg) dose of psilocybin in opaque gelatin capsules were orally consumed at the first and second sessions, respectively. During these sessions, participants lay on a couch in a comfortable, dimly lit room while wearing eyeshades and listening to music (mostly Western art music) through headphones. Throughout these sessions, research personnel remained in the room at all times to respond to participants’ needs, though participants were encouraged to remain in a supine position and direct their attention inward. After each psilocybin session, participants attended follow-up meetings to discuss their experiences.

In order to account for non-treatment-related changes in depression (e.g., interacting with research personnel) and performance on cognitive tasks (i.e., practice effects), the 11 participants in the delayed group completed clinical and cognitive (but not neuroimaging) measurements after screening (pre-delay assessments) and were then monitored weekly through in-person visits and telephone calls during an 8-week delay. At the end of this delay period, participants completed a second set of clinical and cognitive measurements and their first neuroimaging measurements (baseline assessments) followed by the same preparatory, psilocybin, and follow-up sessions administered to the immediate treatment group.

### Clinical and cognitive measures

Depression was assessed with the 17-item GRID version of the HAMD [77] administered by blinded clinician raters via telephone at screening, 1 week post-treatment, and 4 weeks post-treatment. In the delayed control group, the GRID-HAMD was also assessed after 5 and 8 weeks into the delay period.

Notable effects of psilocybin therapy were limited to the Penn Conditional Exclusion Test (PCET; [78]), and descriptions and analyses of the other two tasks can be found in the Supplementary Information (SI; Table S1). The PCET is a set-shifting measure of cognitive flexibility modeled after the Wisconsin Card Sorting Task [79], but the PCET has multiple validated alternate test forms with different stimuli to support repeated measurements. Participants completed a different test form at every visit, and the order of these test forms was counterbalanced across participants. On each trial of the PCET, four figures were simultaneously presented, and participants were required to select the figure that was different from the other three based on one of three criteria (Version 1: line thickness, shape, size; Version 2: location of shape within stimulus box, color, type of shape within stimulus box; Version 3: angle of lines within stimulus box, dashed versus solid lines within stimulus box, and presence or absence of a border on stimulus box; Version 4: sharp or rounded edges, angle of stimulus box, filled or empty stimulus box). Once a figure was selected, participants were presented with immediate feedback (“correct” or “incorrect”). After 10 consecutive correct trials or after 48 total trials, the criterion for a correct response changed until another 10 consecutive correct trials or 48 total trials was reached after which the criterion was changed one last time (followed by a final round of 10 consecutive correct or 48 total trials). Perseverative errors were defined as the number of instances in which three incorrect responses are made based on a previous rule, and they are thought to reflect less cognitive flexibility (or cognitive rigidity). See SI for descriptions and data of other responses. One participant (female, delayed group) refused to complete cognitive tasks, and another participant (female, immediate group) was excluded for looking up the rules to the PCET after the first test (resulting in *N* = 22). Due to a technical error, data from 4 weeks post-treatment was missing from an additional participant (female, delayed group).

### Imaging parameters and preprocessing

Approximately 4 weeks before the first psilocybin session and 1 week after the second psilocybin session (on the same days as cognitive testing) participants were scanned at 7 T (Philips Achieva, Best, The Netherlands) using a 32-channel head coil (Nova Medical, Wilmington, MA). Measurements at each scanning session included a T1-weighted structural MPRAGE (TR/TE = 4100/1.86 ms, flip angle = 7°, acquisition matrix = 220 × 220 mm, voxel size = 1 mm^3^), an echo-planar scan with an emotional processing task (to be reported elsewhere), an echo-planar scan to measure task-free (eyes open) blood-oxygenation level-dependent (BOLD) fMRI (355 TRs, TR/TE = 2000/22 ms, flip angle = 60°, acquisition matrix = 192 × 192 mm, in-plane resolution = 2.5 × 2.5 mm, slice thickness = 3 mm, SENSE acceleration factor = 3, 54 axial-oblique slices parallel to the anterior-posterior commissure line), and stimulated echo acquisition mode (STEAM) short-TE ^1^H-MRS scans (TR/TE/TM = 3000/33/14, acquisition time = 6 min 30 s, NT = 128, bandwidth = 5000 Hz, VAPOR water suppression; water-unsuppressed spectra were acquired with similar parameters and NT = 2) of the ACC (voxel size = 30 × 20 × 20 mm^3^), left hippocampus (voxel size = 35 × 15 × 15 mm^3^), and right hippocampus (voxel size = 35 × 15 × 15 mm^3^; see Fig. S1 for example of spectroscopic voxel placement). Three participants (one male, all immediate group) did not complete at least one imaging session, and one participant (male, delayed group) did not complete a resting state scan. In addition, one participant (male, delayed group), three participants (one male, delayed group and two females, immediate group), and two participants (both females, one immediate group) had poor signal (spectral abnormalities, signal-to-noise <5, or a Cramer-Rao Lower Bound standard deviation >20 for glutamate or NAA) in at least one of their ACC, left hippocampus, and right hippocampus MRS scans, respectively. These participants were, therefore, excluded from their respective analyses (resulting in *N* = 20, 20, 18, and 19 for resting state, ACC, left hippocampus, and right hippocampus scans, respectively).

Spatial preprocessing of functional images was performed in SPM12 and included realignment (motion correction), co-registration of the second BOLD scan with the first BOLD scan, and normalization of BOLD scans to an EPI template in MNI space [80] using a 4th degree B-Spline interpolation. Temporal preprocessing was performed using tools from the Cognitive and Affective Neuroscience Lab (http://github.com/canlab) and included simultaneous bandpass filtering (0.009–0.08 Hz) and nuisance regression. Nuisance parameters consisted of linear trend, the first 5 principal components of voxels containing cerebrospinal fluid and the first 5 principal components of voxels containing white matter signal (both identified using masks derived from segmented, co-registered, and normalized T1-weighted structural images; [81]), 24 motion parameters from realignment (translations and rotations, their derivatives, and squares of all of these; [82]), and motion censoring or “scrubbing” regressors [83] generated from the ART toolbox using outlier detection and intermediate settings (global-signal *z*-value threshold = 5, subject-motion threshold = 0.9 mm). Finally, these data were parcellated by averaging the BOLD signal at each TR across voxels within a 10-mm sphere around each of the 264 vertices of the Power atlas [84], producing 264 timeseries of nodes. Susceptibility artifacts due to 7 T magnetic field inhomogeneities at tissue boundaries led to variable degrees of signal-dropout across participants. On average across scans, 59% of voxels had acceptable signal-to-noise. However, only 89 Power atlas nodes had acceptable signal-to-noise in at least 75% of within-sphere voxels across all scans, and thus, functional connectivity data were only analyzed for these 89 ROIs (see Fig. S2 for maps of acceptable signal). Increasing the threshold for allowing nodes into analyses did not qualitatively change results (SI). The split-half reliability of functional connectivity of these 89 nodes was high, and head motion after preprocessing was unrelated to sFC and dFC (Figs. S3–S4).

MRS data were visually inspected to detect any spurious artifact or lipid contamination. Afterwards, spectra were preprocessed using an in-house developed software based on FID-A [85] and quantified with LCModel 6.3 [86] modeling the spectrum from 0.5 to 4.2 ppm. Basis sets consisted of 20 metabolites generated using custom-built fully localized density matrix simulations [87]. Macromolecules were simulated in LCModel (NSIMUL = 12). Signal of total creatine was used as an internal standard. Spectra showed overall good spectral quality, though spectra from the left and right hippocampi showed higher variability, especially in the 4–3 ppm region (Fig. S5 and Table S2).

### Analyses

Univariate analyses consisted of one-way ANOVAs and two-tailed *t* tests comparing clinical, cognitive, and neural baseline measures that occurred ~2 weeks before the first psilocybin session to those measures post-psilocybin therapy (i.e., 1 week and 4 weeks post-treatment). To measure potential non-treatment-related effects of multiple clinical and cognitive tests, in the delayed group, we compared pre-delay measures to the baseline timepoint 2 weeks before psilocybin therapy and supported null effects with Bayes factors. Exploratory correlations were calculated using Pearson’s correlation coefficients.

For analysis of MRS data, we focused on glutamate and NAA, as these are commonly measured metabolites that have reliable spectra, are altered in depression and from antidepressant treatments [74, 75], and have been found to be sensitive to the effects of psychedelics [49]. For the BOLD data, matrices of sFC and dFC were created for each participant and experimental condition (Fig. S6). For sFC, the Pearson’s *r* between the timeseries of all pairwise combinations of 89 nodes was computed, producing 3916 functional connections (edges) with all *r*-values Fisher *z*-transformed for analysis. For dFC, correlation timeseries were first computed for each edge using dynamic conditional correlations (DCC; [88]), a more reliable method than commonly used sliding window approaches [89]. dFC matrices were then computed by calculating the variance of each correlation timeseries. As described previously, we focused our analyses on the ACC and PCC based on their roles in cognitive flexibility and psychedelic drug action. The specific ACC node was selected based on the Power atlas node contained within the most MRS scans of the ACC. The specific PCC node was selected based on the results of a prior study that found increased sFC between the subgenual cingulate and a region corresponding to the left PCC/precuneus [53].

In order to test whether baseline measures of sFC and dFC predicted post-treatment changes in depression and cognitive flexibility, we constructed and tested the performance of several connectome-based predictive models [90–92]. This approach selects neural features (i.e., baseline sFC and dFC edges) most correlated with behavioral variables (i.e., changes in GRID-HAMD scores and PCET perseverative errors), trains a linear model from a subset of these data by summing these features, and tests these models by applying them to predict behavior from brain data left out of training. We implemented leave-one-participant-out cross-validation and inspected the relationship between observed and predicted data across folds (Pearson’s *r*). Features are selected if their correlation with behavior surpasses a thresholded level of significance (typically *p* < 0.010). Because of the relatively small *N* and number of edges from which to select, we report the robustness of different models across a range of thresholds for feature selection (*p* < 0.010–0.050).

## Results

### Enduring effects of psilocybin therapy on depression and cognitive flexibility

**Fig. 1:**
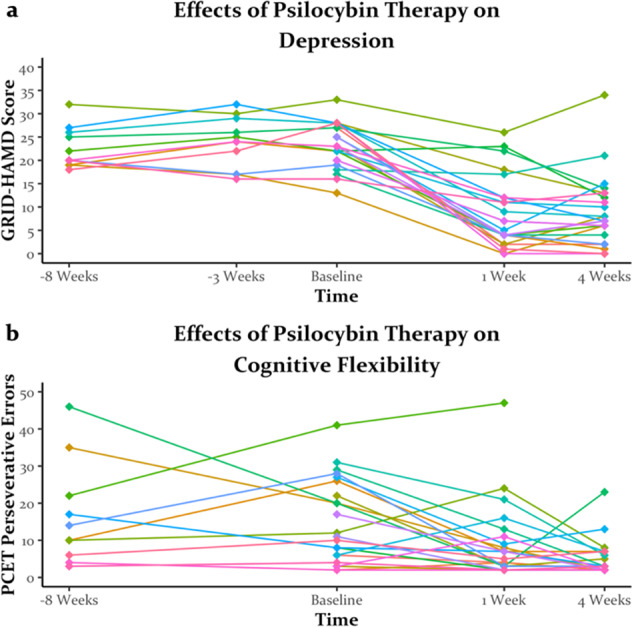
The effects of psilocybin therapy on depression and cognitive flexibility. Depression symptomology (**a**) as measured by the GRID-Hamilton Depression Rating Scale (GRID-HAMD; also reported in [3]) and cognitive flexibility (**b**) as measured by perseverative errors on the Penn Conditional Exclusion Test (PCET) were improved from pre- to post-psilocybin therapy. These changes were not found between repeated tests pre-psilocybin therapy in the delayed group (i.e., between −8 weeks and Baseline time points). Each line color represents a unique participant, and the mapping of individual colors to unique participants remains consistent across all figures in which individual participant data are plotted.

As reported previously [3] and shown in Fig. 1a, psilocybin therapy robustly decreased GRID-HAMD scores in nearly every participant from baseline to 1 week and 4 weeks post-treatment. This reduction in depression was supported by a massive effect of timepoint (*F*(2, 46) = 69.95, *p* < 0.001, $\eta _P^2$ = 0.75). There was moderate evidence supporting the null hypothesis that GRID-HAMD scores did not change between the pre-delay and baseline timepoint in the delayed group before they received psilocybin (*F*(2, 20) = 0.25, *p* > 0.250, BF = 0.23).

Similar to GRID-HAMD scores, perseverative errors on the PCET generally decreased from baseline to 1 week post-psilocybin therapy (Fig. 1b) with this effect sustained 4 weeks post-treatment. This improvement in cognitive flexibility was supported by a main effect of timepoint (*F*(2, 40) = 10.90, *p* < 0.001, $\eta _P^2$ = 0.35). The effect of psilocybin therapy on PCET perseverative errors was unlikely to be explained by practice effects, as there was moderate evidence supporting the null hypothesis that perseverative errors did not change in the delayed group prior to receiving psilocybin (95% CI = [−9.77, 10.57], *t*(9) = 0.09, *p* > 0.250, BF = 0.31). Changes in GRID-HAMD scores were not correlated with changes in PCET perseverative errors across any set of timepoints (all |*r* | s < 0.2, all *p*s > 0.250).

### Enduring effects of Psilocybin therapy on brain function

**Fig. 2:**
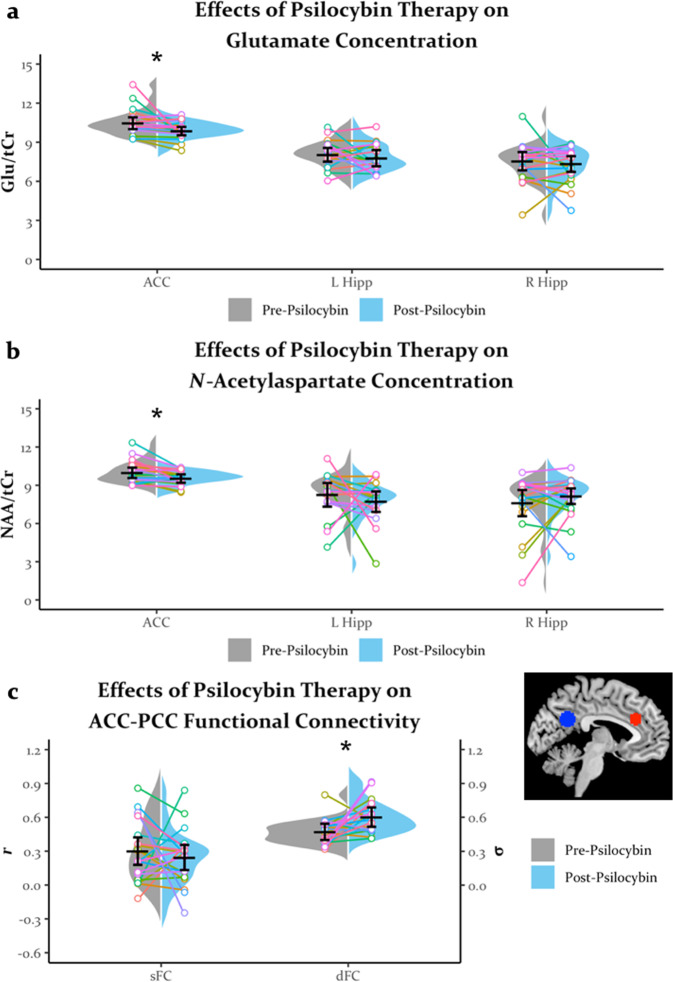
The effects of psilocybin therapy on neurometabolite concentrations and functional connectivity. Split violin plots with horizontal bars reflecting the mean, error bars reflecting the 95% confidence interval, and “strings” reflecting individual participant data. The color of a given participant is consistent across all figures plotting individual participant data. Psilocybin therapy reduced (**a**) glutamate and (**b**) *N*-acetylaspartate in the anterior cingulate cortex but not in the left or right hippocampus. **c** Psilocybin therapy increased dFC but not sFC between the anterior and posterior cingulate. *p < 0.050. ACC anterior cingulate cortex, L Hipp left hippocampus, R Hipp right hippocampus, Glu glutamate, tCr total creatine, NAA *N*-acetylaspartate, PCC posterior cingulate.

Both glutamate (95% CI = [0.11, 1.10], *t*(19) = 2.54, *p* = 0.020, *d* = 0.57) and NAA (*t*(19) = 3.05, *p* = 0.007, *d* = 0.68) were decreased in the ACC 1 week after psilocybin therapy (Fig. 2a, b). Decreases in ACC neurometabolite concentrations were regionally selective, as there were no significant changes in glutamate and NAA in the left or right hippocampi (all *t*s < 1.04, all *p*s > 0.250). All correlations between changes in glutamate or NAA and changes in GRID-HAMD scores or PCET perseverative errors were non-significant (all |*r* | s < .36, all *p*s > 0.130).

**Fig. 3:**
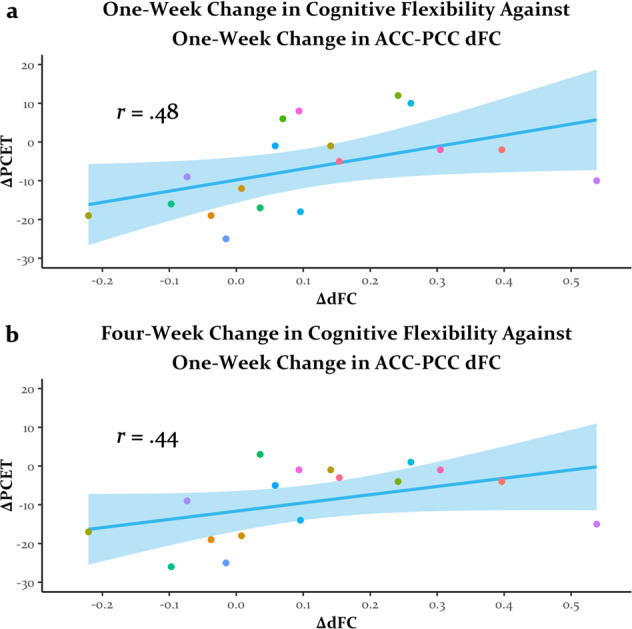
Greater pre- to post-psilocybin therapy increases in neural flexibility were associated with less improvements in cognitive flexibility. Relationship between changes in anterior to posterior cingulate cortex (ACC and PCC, respectively) dynamics of functional connectivity (dFC) 1 week post-psilocybin therapy and changes in perseverative errors on the Penn Conditional Exclusion Test (PCET) at (**a**) 1 week and (**b**) 4 weeks post-psilocybin therapy.

In general, sFC numerically decreased and dFC numerically increased across the brain after psilocybin therapy (Fig. S7). Prior work found increases in sFC between the ACC and PCC 1 day after ayahuasca administration [51]. Therefore, we examined the effects of psilocybin therapy on ACC-PCC functional connectivity. Although we observed no significant change in sFC (95% CI = [−0.12, 0.23]; *t*(19) = 0.67, *p* > 0.250), there was a fairly reliable increase in dFC between the ACC and PCC 1 week after psilocybin therapy (95% CI = [0.02, 0.21]; *t*(19) = 2.87, *p* = 0.010, *d* = 0.64; Fig. 2c). Exploratory correlations found a moderate association between these increases in dFC and decreases in PCET perseverative errors 1 week post-psilocybin therapy (*r*(16) = 0.48, *p* = 0.043; Fig. 3a) with a slightly smaller non-significant correlation observed at 4 weeks post-psilocybin therapy (*r*(16) = 0.44, *p* = 0.079; Fig. 3b). Interestingly, these correlations were positive, suggesting that an increase in dFC may actually be associated with less of a decrease in perseverative errors (i.e., less increase in cognitive flexibility). All changes in ACC-PCC functional connectivity and changes in GRID-HAMD scores were non-significant (all |*r*|s < 0.10, all *p*s > 0.250).

### Prediction of improvement from baseline functional connectivity

**Fig. 4:**
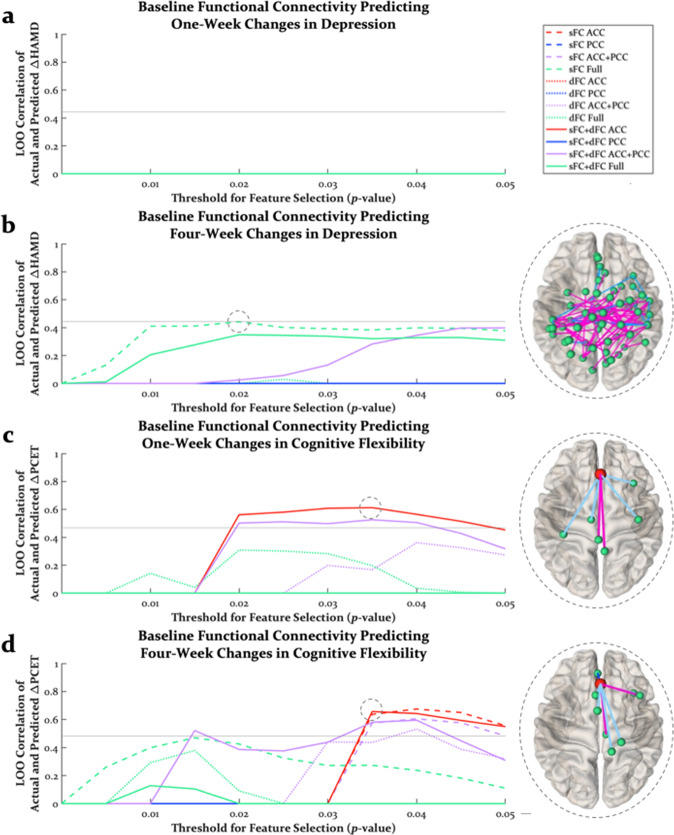
Connectome-based predictive modeling of pre- to post-psilocybin therapy changes in depression and cognitive flexibility. Performance of models trained on baseline functional connectivity as a function of the threshold for feature selection predicting 1-week (**a**) and 4-week (**b**) changes in depression (ΔHAMD) and 1-week (**c**) and 4-week (**d**) changes in cognitive flexibility (ΔPCET). Horizontal gray lines indicate model performance at *p* = 0.05. Brains on the right side of panels are the best-performing models (see gray circles on performance lines). A brain was not plotted for predicting 1-week changes in depression, as there was no model was that performed at *r* > 0. Edges that were included in at least 75% of folds were plotted in dark blue, magenta, and light blue, representing sFC edges positively correlated with behavior, sFC edges negatively correlated behavior, and dFC edges positively correlated with behavior, respectively. Brain visualizations created with NeuroMArVL (https://immersive.erc.monash.edu/neuromarvl/). LOO leave-one-out, sFC static functional connectivity, dFC dynamics of functional connectivity, ACC anterior cingulate cortex, PCC posterior cingulate cortex.

A goal of clinical neuroimaging is to use tools from cognitive neuroscience to identify biomarkers to inform the diagnosis and treatment of psychiatric disorders. In order to explore such possibilities and further elucidate the counterintuitive findings that greater psilocybin-induced neural changes can be associated with less improvement in cognitive flexibility, we trained connectome-based predictive models on baseline functional connectivity to predict changes in depression and cognitive flexibility at 1 and 4 weeks post-psilocybin therapy. Models for each behavioral variable were trained on baseline sFC, dFC, and both sFC and dFC of the ACC, PCC, both ACC and PCC, and the connectome of 3916 edges (the “full” connectome). Performance of these models across a range of thresholds for feature selection is shown in Fig. 4.

There are several points worth noting regarding these predictive models. No model was capable of predicting 1-week improvements in depression (Fig. 4a). In contrast, the prediction of 4-week improvements in depression was moderately good and consistent across thresholds for feature selection using the model trained on baseline sFC of the full connectomre (Fig. 4b). Successful prediction of 1-week and 4-week changes in cognitive flexibility was best with both baseline sFC and dFC of the ACC with the addition of edges emanating from the PCC reducing model performance (Fig. 4c, d), though both models contained edges from the ACC to posteromedial regions. These models predicting changes in cognitive flexibility were fairly robust to relaxing the threshold for edge selection.

**Fig. 5:**
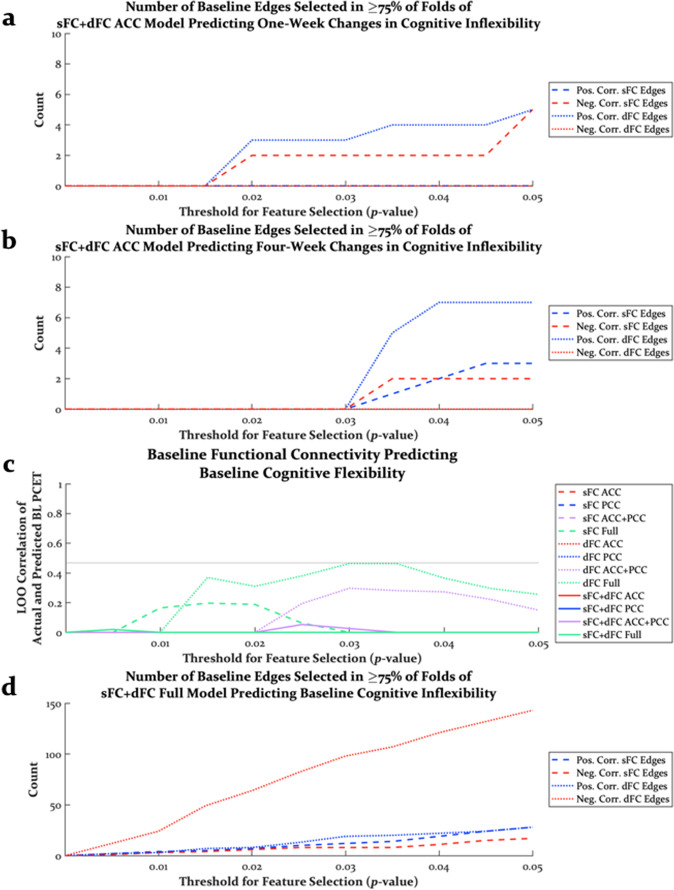
The relationship between model features and cognitive flexibility. Positively and negatively correlated features that were selected in the best models trained on baseline sFC and dFC predicting (**a**) 1-week changes and (**b**) 4-week changes in cognitive flexibility. Positively correlated dFC edges were consistently selected in these models, suggesting that greater baseline dFC was associated with more PCET perseverative errors (i.e., greater cognitive rigidity). In contrast, when models were trained on baseline dFC to predict baseline cognitive flexibility (**c**), dFC edges were still predictive of cognitive flexibility, but the correlations between dFC edges and cognitive flexibility was reversed (**d**). The sFC + dFC Full model was plotted here to highlight that regardless of how many edges were allowed into the model, far more dFC edges were negatively correlated with PCET perseverative errors. Pos. positively, Neg. negatively, Corr. correlated, LOO leave-one-out, sFC static functional connectivity, dFC dynamics of functional connectivity, ACC anterior cingulate cortex, PCC posterior cingulate cortex.

To further interrogate predictive model features, we examined the relationship between individual model features (sFC and dFC edges) and behavioral variables across thresholds for feature selection. Whereas baseline sFC and dFC edges predicting 4-week improvements in depression could be both positively and negatively correlated with changes in depression (Fig. S8), the best models predicting 1-week and 4-week changes in cognitive flexibility (i.e., sFC and dFC of the ACC) contained features that were counterintuitively related to behavior. Specifically, baseline dFC edges were selected more often than sFC edges, and these dFC edges were positively correlated with the reductions in PCET perseverative errors (Fig. 5a, b). In contrast, when models were trained on baseline functional connectivity to predict baseline cognitive flexibility (Fig. 5c), again more dFC edges were selected than sFC edges, but these brain-behavior correlations were strikingly reversed (Fig. 5d). That is, at baseline, greater dFC was predictive of greater cognitive flexibility (less PCET perseverative errors), but after psilocybin therapy, which tended to increase brain-wide dFC, greater baseline dFC was associated with less improvement in cognitive flexibility. Similar to models predicting changes in depression, models trained on baseline functional connectivity to predict baseline depression involved sFC and dFC that were both positively and negatively correlated with depression (Fig. S8). Finally, training predictive models only on nodes with acceptable signal-to-noise in 100% of within-sphere voxels across all scans produced qualitatively similar results that did not change the interpretation of these results (Figs. S9–S11).

## Discussion

Psilocybin therapy was shown to increase cognitive and neural flexibility in patients with MDD. Increases in cognitive flexibility were selective, as tasks measuring response inhibition, selective attention, and abstract reasoning were not impacted. Although a lack of correlation between improvements in cognitive flexibility and improvements in depression might suggest that cognitive flexibility is not mechanistically related to antidepressant effects of psychedelic therapy, the GRID-HAMD assesses different features of current depression (e.g., affect and appetite) and not necessarily cognitive impairments that may pre-date treatment seeking [13] or that persist in those who otherwise respond to treatment [28, 29]. While improved cognitive flexibility itself is a noteworthy and important effect of psilocybin therapy, it is possible that enhancements in cognitive and neural flexibility may open a window of plasticity [56] during which improvements can be facilitated (e.g., with supportive psychotherapy), though this remains speculative. In the current sample, despite psilocybin therapy generally increasing dFC across the brain, the benefits of this neural flexibility were nonlinear. That is, larger pre- to post-treatment increases in dFC between the ACC and PCC and greater baseline dFC of the ACC was associated with less improvement in cognitive flexibility. An implication of this finding is that sub-populations of patients (i.e., those with lower baseline neural flexibility) may be more likely to benefit from psychedelic therapy.

This study had several limitations. The study design was not placebo-controlled and instead utilized a pre- vs. post-treatment design, suggesting that the observed effects could be attributable to expectancy, practice, or exposure effects. We believe this is an insufficient explanation, as the effects of psilocybin therapy on depression were far larger than typical placebo effects [93, 94], and there was no evidence for practice effects on the PCET in the delayed group that performed the task twice before receiving psilocybin therapy. Moreover, it seems implausible for certain biological signals to be decreased by placebo effects such as ACC glutamate, which has good test-retest reliability [95]. Nevertheless, in a recent double-blind clinical trial on the treatment of MDD comparing psilocybin to the SSRI citalopram using equivalent protocols for psychotherapy in both conditions [7], citalopram was found to be effective, though somewhat less so than psilocybin, 1 week after treatment, much sooner than is typically expected with a SSRI. Another study found that a trivial dose of psilocybin in the context of the psychotherapy that accompanies psilocybin therapy had considerable reductions on depression [4]. Thus, one could reasonably interpret that large doses of psychotherapy and expectancy of receiving psilocybin provide for a substantial therapeutic element of psilocybin therapy. Another limitation to this study was that due to signal dropout at 7 T, we unfortunately lost a large portion of brain regions, though including more or less noisy regions into analyses did not qualitatively change our results. In addition, the relatively few edges of the ACC-based predictive model of cognitive flexibility might actually speak to its robustness because there were likely missing edges that include other regions known to support cognitive flexibility (e.g., orbitofrontal cortex; [8]). However, signal dropout in various regions implicated in the pathophysiology of mood disorders (e.g., the hippocampus [26]) may have precluded finding better-performing models to predict changes in depression after psilocybin therapy. Considering the heterogeneity of depression [96], such better-predicting models may rely on a more distributed network of brain regions than we were able to sample within our data. Finally, the generalizability of predictive models is expected to vary as a function of sample size. The current findings are based on a relatively small sample size for predictive models, though a slightly larger sample (*N* = 25) was previously used to build a predictive model of attention that has consistently generalized to other measures of attention, clinical symptoms, and acute drug effects [97–99].

Our findings support a role of the ACC, as well as other parts of the cingulate gyrus, in the enduring effects of psilocybin on cognition. Consistent with past work that found changes in interactions between the ACC and PCC [51] and the subgenual cingulate and PCC [53] 1 day after psychedelic administration, we found increased dFC between the ACC and PCC. Moreover, models trained only on ACC functional connectivity to predict cognitive flexibility outperformed all other models, including those that could select features from the full connectome, which had over 40 times the number of edges than a single node. Finally, the selective decreases in both glutamate and NAA of the ACC suggest a reduction in neural metabolism, though further work will be needed to determine how such reductions in metabolism relate to increased neural flexibility.

The possibility that greater neural flexibility might attenuate the benefits of psilocybin therapy to cognitive flexibility should perhaps not be surprising. Patients with schizophrenia, for example, exhibit increased brain wide neural flexibility [67]. It could be that psilocybin pushes neural flexibility in some individuals past the zone of largest therapeutic efficacy. One question that our study was not designed to address is whether such detrimental effects are due to too much neural flexibility or too enduring of elevations in neural flexibility. Neural flexibility could be required during the acute effects of psychedelics to permit the exploration of novel cognitive states that can allow one to escape maladaptive attractor basins (e.g., rumination). Perhaps even in the days following the acute effects, some neural flexibility may be needed for the integration process, allowing for greater responsiveness to continuing psychotherapy. However, persisting increases in neural flexibility could become destabilizing to an individual’s life, resulting in, for example, lower attention [90, 100]. It may be that psychedelics with longer or shorter enduring effects are more beneficial for different psychiatric disorders. Overall, our work suggests that psilocybin therapy may improve cognitive flexibility in psychiatric illness, but it highlights potential boundary conditions of psychedelic-induced neural flexibility and its relationship to cognitive improvements.

## Supplementary information

Supplemental Material

## Data Availability

Computed code used to analyze data or generate figures can be requested from MKD.
