# Supplementary material for: Psilocybin therapy increases cognitive and neural flexibility in patients with major depressive disorder
Source: Transl Psychiatry. 2021 Nov 8;11:574. doi: 10.1038/s41398-021-01706-y (PMC8575795; doi:10.1038/s41398-021-01706-y)
Supplement: Supplementary file 1 — Supplemental Material [file 41398_2021_1706_MOESM1_ESM.docx]

**Supplementary Information for**

**Psilocybin Therapy Increases Cognitive and Neural Flexibility in**

**Patients with Major Depressive Disorder**

Manoj K. Doss, PhD, Michal Považan, PhD, Monica D. Rosenberg, PhD, Nathan D. Sepeda, B.A., Alan K. Davis, PhD, Patrick H. Finan, PhD, Gwen S. Smith, PhD, James J. Pekar, PhD, Peter B. Barker, DPhil, Roland R. Griffiths, PhD, & Frederick S. Barrett, PhD

**Methods**

*Penn Conditional Exclusion Test*

Measures other than the primary outcome, perseverative errors (i.e., three consecutive incorrect responses based on a previous rule) on the Penn Conditional Exclusion Test (PCET; ^1^ include number correct, which can increase with more perseverative errors, and total number of errors, which can be random or due to exploration after a rule change. Median reaction times can also be computed for these measures. See Table 1 for results of these outcomes.

*Stroop Test*

The Stroop task for this study was modeled after the original Stroop paradigm ^2^ and downloaded from the Cognitive Experiments I package (version 6) on the Neurobehavioral Systems Presentation website (https://www.neurobs.com/menu_presentation/menu_teaching/exp_pack?pack_id=1). This version of the Stroop task consisted of a series of trials in two phases: an ink-naming phase and a word-naming phase. In each trial of the ink-naming phase, participants were shown letters in the middle of the screen, printed in one of four possible colors (red, green, blue, or yellow), and the task was to press a button on a keyboard corresponding the color in which letters were printed. Two ink-naming trial types were presented in random order: neutral trials (with “xxx”, “xxxx”, “xxxxx”, or “xxxxxx” printed in a given color), and incongruent trials (with a given color word printed in a color not corresponding to the word). In each trial of the word-naming phase, participants were shown the name of one of four colors (red, green, blue, or yellow) presented in the middle of the screen, printed in one of four different colors, and the task was to press a button on the keyboard corresponding to the color that was named by the word rather than the color in which the word was printed. Two word-naming trial types were presented in random order: neutral trials (printed in the color represented by the word) and incongruent trials (printed in either red, green, blue, or yellow). Each trial type was presented 24 times within each block. Each trial began with the presentation of a fixation cross for 500 ms. Participants were then given five seconds to respond to each stimulus, and feedback (“correct” or “incorrect”) was presented for one second after each response. “Incorrect” was presented for one second if the five second response time elapsed on any given trial. Each trial was separated by a one-second inter-stimulus interval, between the offset of feedback and the beginning of the fixation cross.

Dependent variables on this task include hit rate, reaction time for hits, *d*’, and interference, defined as the difference in reaction times between incongruent and neutral trials. Order effects involving the preceding trial were examined, as these can impact performance ^3^, producing four additional conditions: neutral trials preceded by incongruent trials, incongruent trials preceded by neutral trials, neutral trials preceded by neutral trials, and incongruent trials preceded by incongruent trials. Gratton effects are defined as the difference in reaction times between incongruent trials preceded by neutral trials (i.e., the hardest trial type) and neutral trials preceded by neutral trials (i.e., the easiest trial type). Reaction times were log transformed (log(1/RT)). In addition to the one participant who refused cognitive tasks, two participants (one male and one female, both delayed group) did not complete the Stroop task due to technical errors (resulting in *N* = 21).

*Short Penn Verbal Reasoning Task*

The SPVRT consists of eight verbal analogies with the correct answer amongst a four-alternative forced choice (e.g., Predicament is to carelessness as response is to…1. Answer, 2. Stimulus, 3. Effect, 4. Good; the answer is Stimulus). The number of correct answers and the median reactions times are thought to reflect verbal intellectual ability. In addition to the one participant who refused cognitive tasks, one participants (female, delayed group) did not complete the Short Penn Verbal Reasoning Task (SPVRT; ^4^) one week post-treatment and was therefore excluded (resulting in *N* = 22). Due to a technical error, an additional participant (female, delayed group) did not complete the SPVRT at four weeks post-treatment.

**Figures**

*Magnetic Resonance Spectroscopy Acquisition*


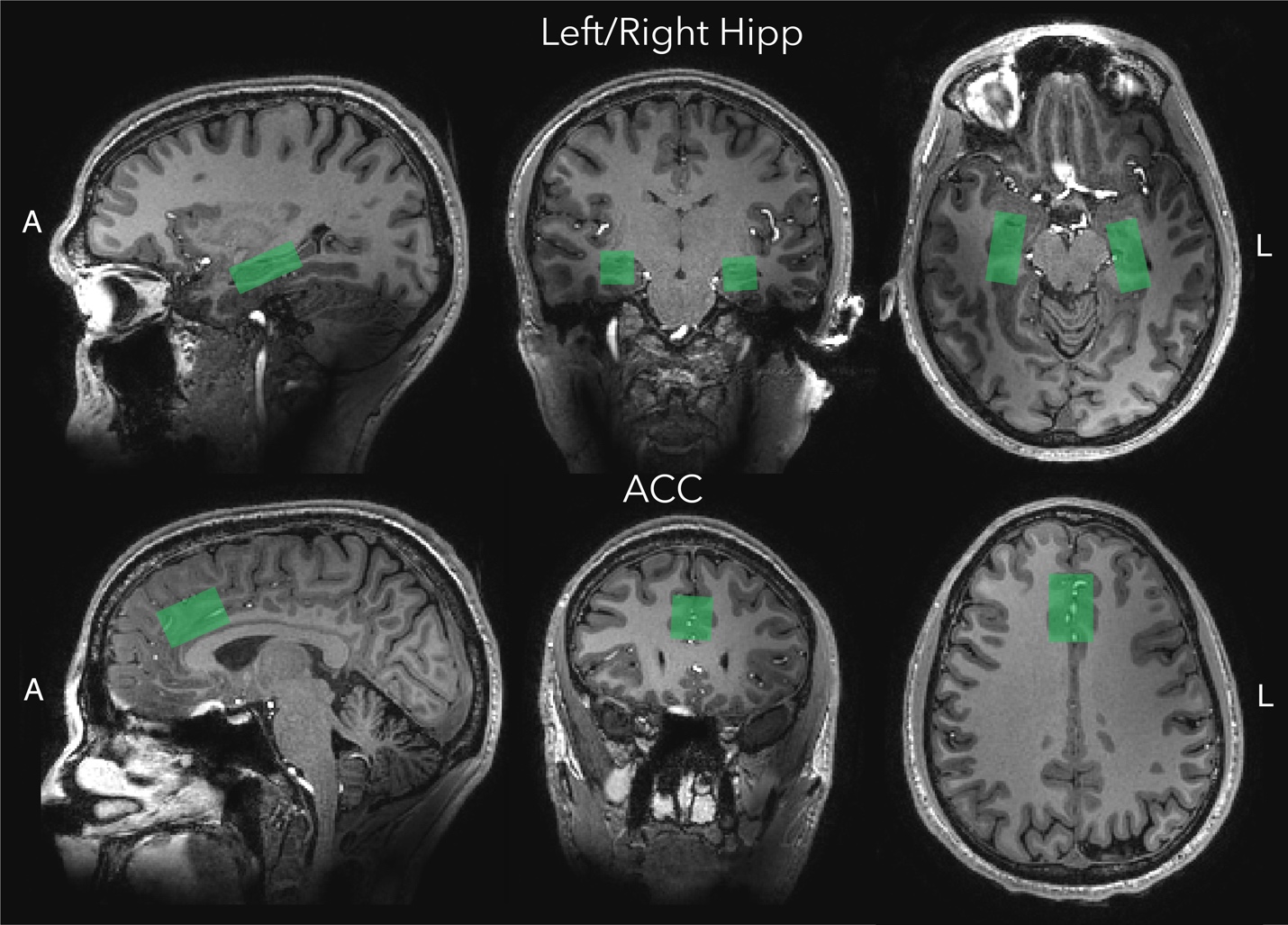


**Figure S1.** Example of the spectroscopic voxel placement (green box) on the T_1_-weighted image in left/right hippocampus (top row; 35 × 15 × 15 mm^3^) and anterior cingulate (bottom row; 30 × 20 × 20 mm^3^). Hipp = hippocampus, ACC = anterior cingulate cortex.

*Signal Dropout in Functional Images*


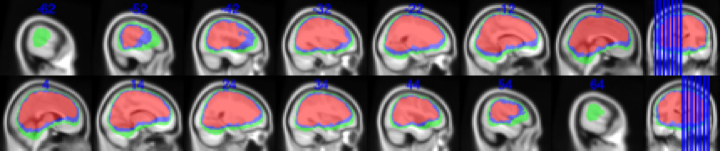


**Figure S2.** Voxels present in at least 50% (green), 85% (blue), and 100% (red) of scans.

*Reliability of First and Second Half of Scans*

As a sanity check, the reliability of static and dynamic functional connectivity (sFC and dFC, respectively) was tested by correlating edges during the first half of a scan with edges during the second half a scan in each subject. Fig. S4 plots every edge of every participant for each measure in each drug condition. In both drug conditions, reliability was high and similar for sFC (pre-psilocybin: *r* = .77; post-psilocybin: *r* = .77) and dFC (pre-psilocybin: *r* = .78; post-psilocybin: *r* = .79).


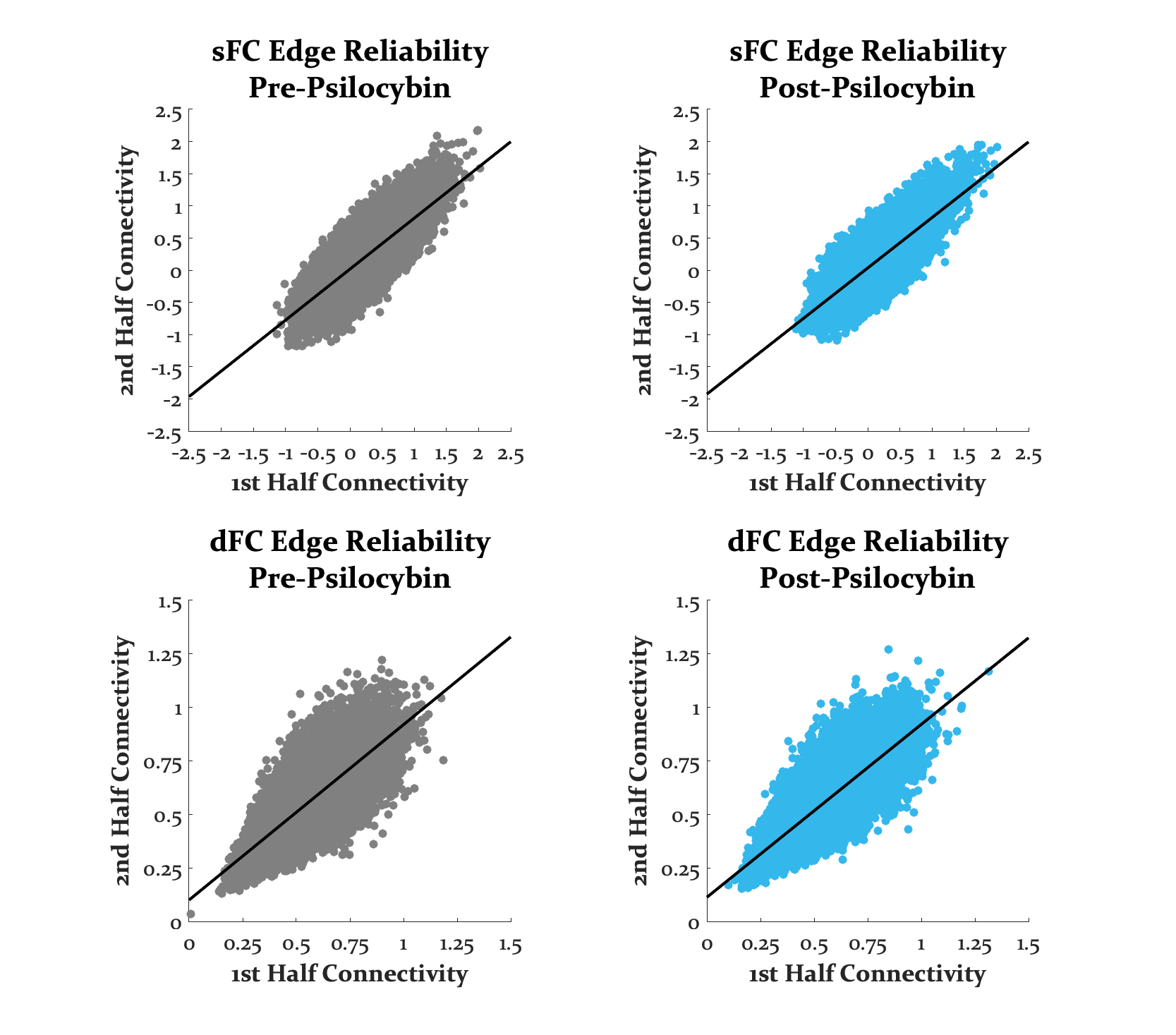


**Figure S3.** Reliability between the first and second half of scans for static (sFC; top two panels) and dynamic (dFC; bottom two panels) functional connectivity for pre- (left two panels) and post-psilocybin (right two panels) scans. Each point represents a single edge for an individual subject.

*Motion Analyses*

Because head movement can be a major confound in fMRI studies, especially with clinical populations exhibiting greater motion ^5^, we compared head motion between the pre- and post-psilocybin scans. Neither framewise displacement (*t*(19) = .90, *p* > .250) nor the number of scrubbed volumes (spikes; *t*(19) = .66, *p* > .250) significantly differed between the two scans. As a further sanity check, we looked at the relationship between internodal distance and the correlation of edgewise functional connectivity and framewise displacement (Figure S5), as motion is known to particularly increase sFC between more distant nodes ^6^. The correlations between internodal distance with the correlation of sFC and framewise displacement were low and similar between pre- and post-psilocybin scans (pre-psilocybin: *r* = .04; post-psilocybin: *r* = .05). Correlations between internodal distance with the correlation of dFC and framewise displacement were also low and similar between pre- and post-psilocybin scans (pre-psilocybin: *r* = .05; post-psilocybin: *r* = -.03).

**
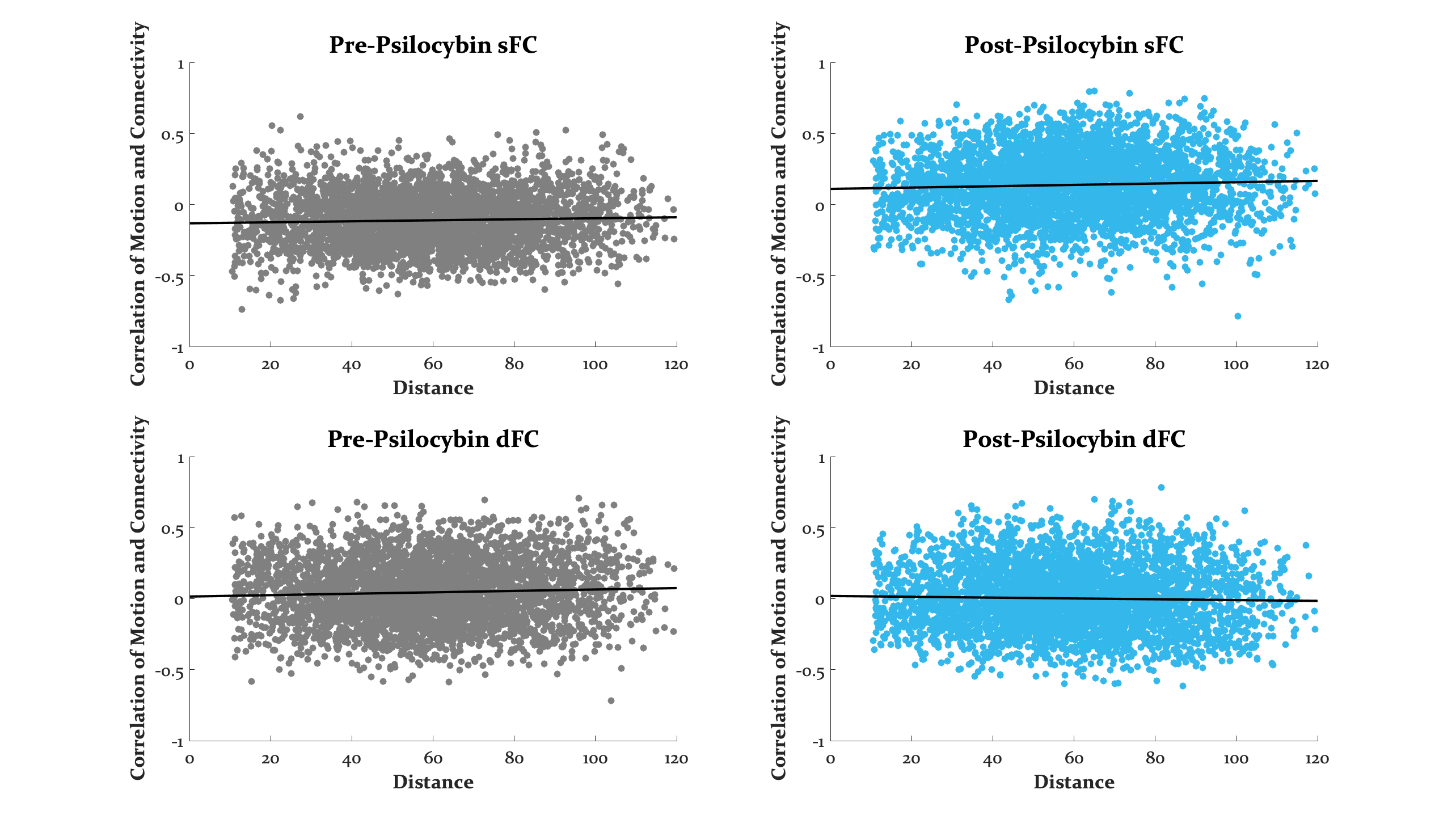
**

**Figure S4.** Relationship between internodal distance and the modulation of static (sFC; top two panels) and dynamic (dFC; bottom two panels) functional connectivity by motion. This relationship was negligible and similar between pre- (left two panels) and post-psilocybin (right two panels), suggesting that changes in functional connectivity between drug conditions was not related to motion.

*Spectra Quality*


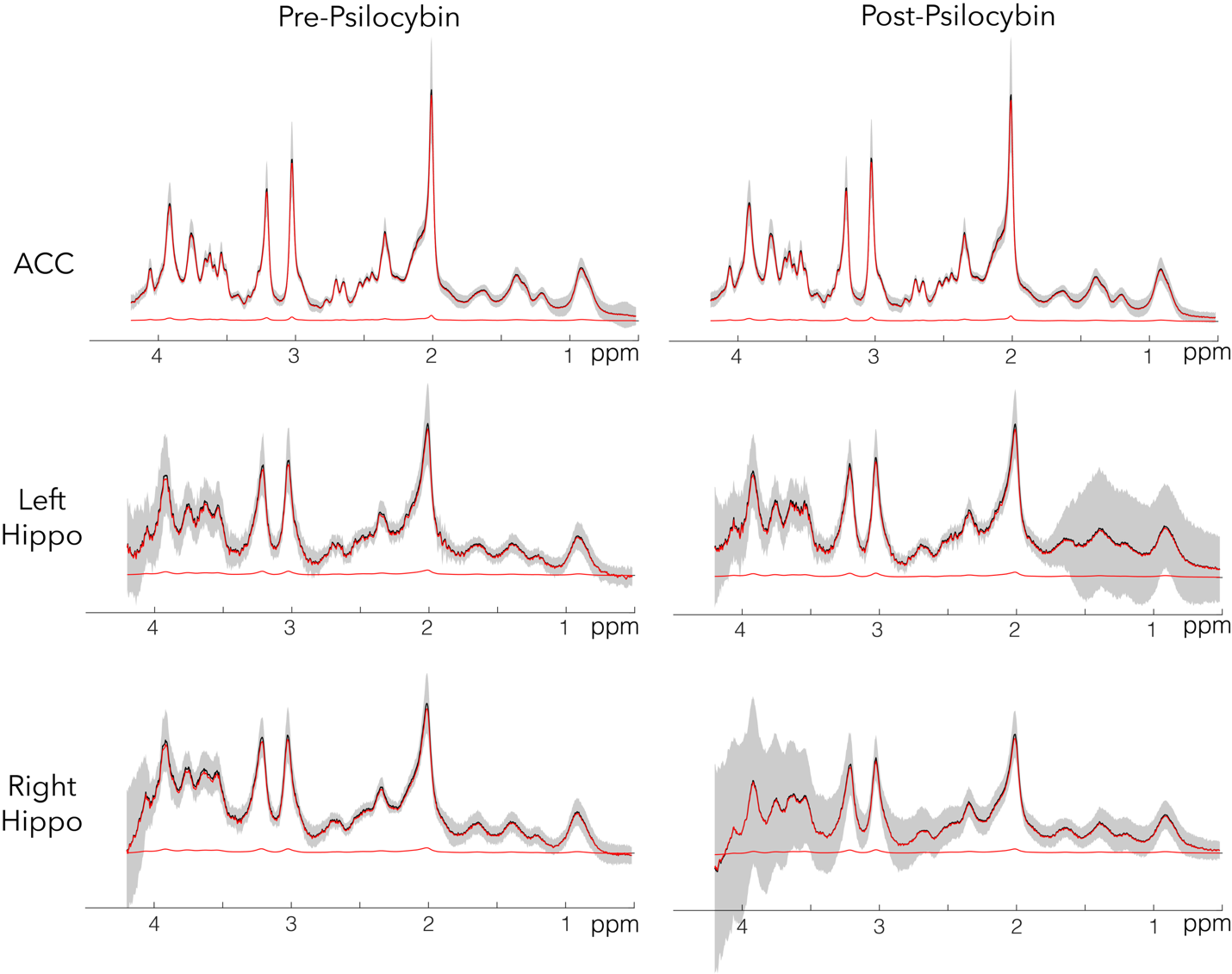


**Figure S5.** Average spectra (black) from the anterior cingulate cortex and the left and right hippocampi are shown separately for pre-psilocybin and post-psilocybin timepoint with average LCModel fit results (red) and average residuals (red, below spectrum). Gray areas represent one standard deviation. ACC = anterior cingulate cortex, Hipp = hippocampus.

*Functional Connectivity Matrices*


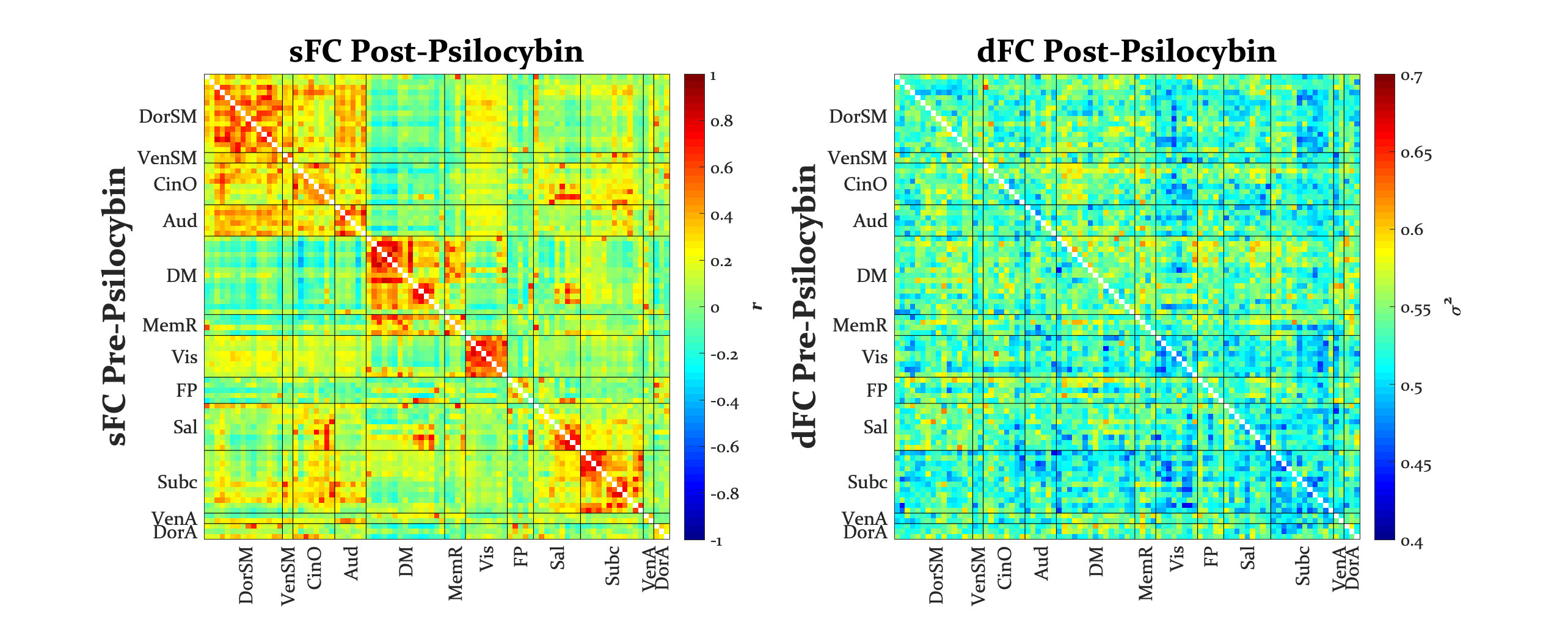


**Figure S6.** Static (sFC; left) and dynamic (dFC; right) functional connectivity matrices for all pairwise functional connections (89 nodes, 3,916 edges) from the pre- and post-psilocybin scans, averaged across participants. Each row and each column represent a single node as defined by the Power functional brain atlas, and the lower and upper triangles represent pre- and post-psilocybin scans, respectively. The color of each off-diagonal cell represents the Pearson correlation (*r*) of variance (σ) for an edge’s static and dynamic connectivity, respectively.


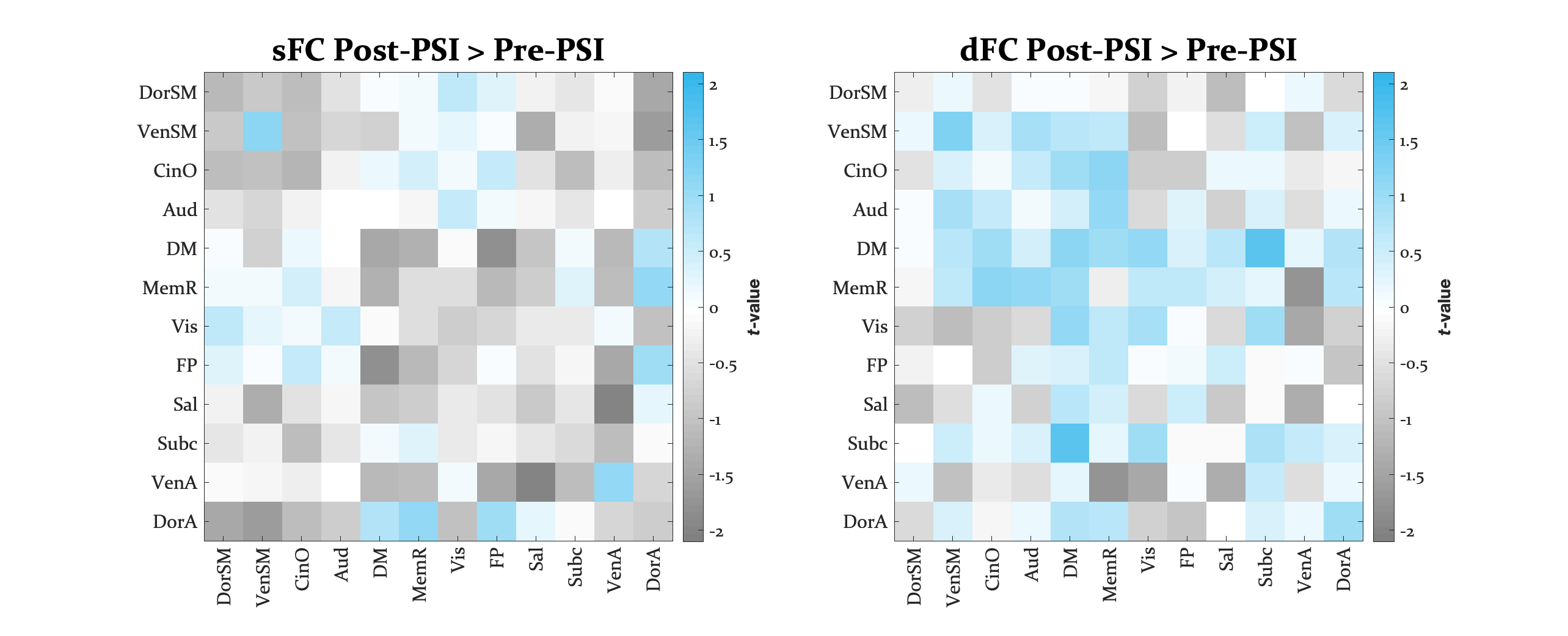


**Figure S7.** Differences (*t*-values) in static functional connectivity (sFC) and dynamics of functional connectivity (dFC) within- and between-networks for pre- vs. post-psilocybin therapy (Pre-PSI, and Post-PSI, respectively). Each row and column represent a single brain network as defined by the Power functional brain atlas. The diagonal and off-diagonal cells represent differences in within- and between-network connectivity, respectively. Although interpretations of changes in network function should be interpreted with caution, as these networks were far from complete due to signal dropout, they simply highlight that sFC generally decreased across the brain, whereas dFC generally increased across the brain.

*Additional Connectome-Based Predictive Models*


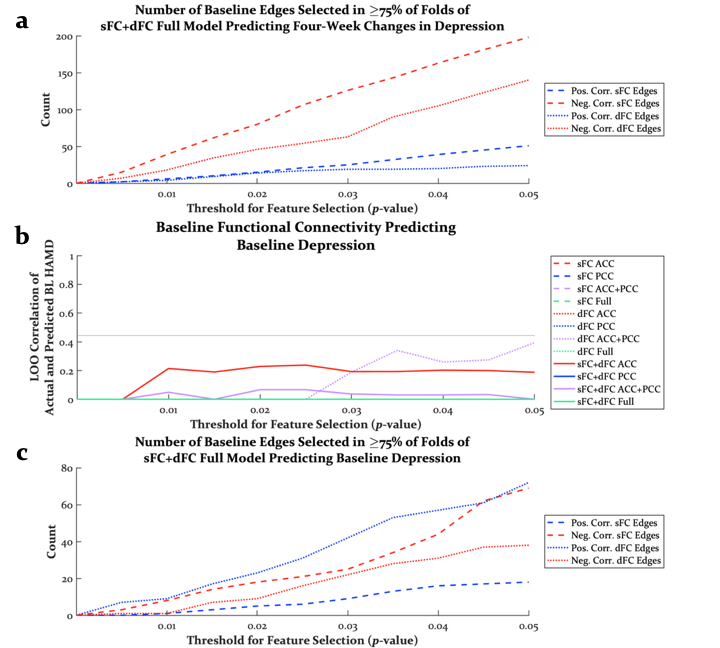


**Figure S8.** **(a)** The number of baseline sFC and dFC edges positively and negatively correlated with four-week changes in depression. **(b)** Performance of models trained on baseline functional connectivity as a function of the threshold for feature selection predicting predicting baseline depression (HAMD). Horizontal grey line indicates model performance at *p* = .05. **(c)** The number of baseline sFC and dFC edges positively and negatively correlated with baseline depression. Pos. = positively, Neg. = negatively, Corr. = correlated, LOO = leave-one-out, sFC = static functional connectivity, dFC = dynamics of functional connectivity, ACC = anterior cingulate cortex, PCC = posterior cingulate cortex.


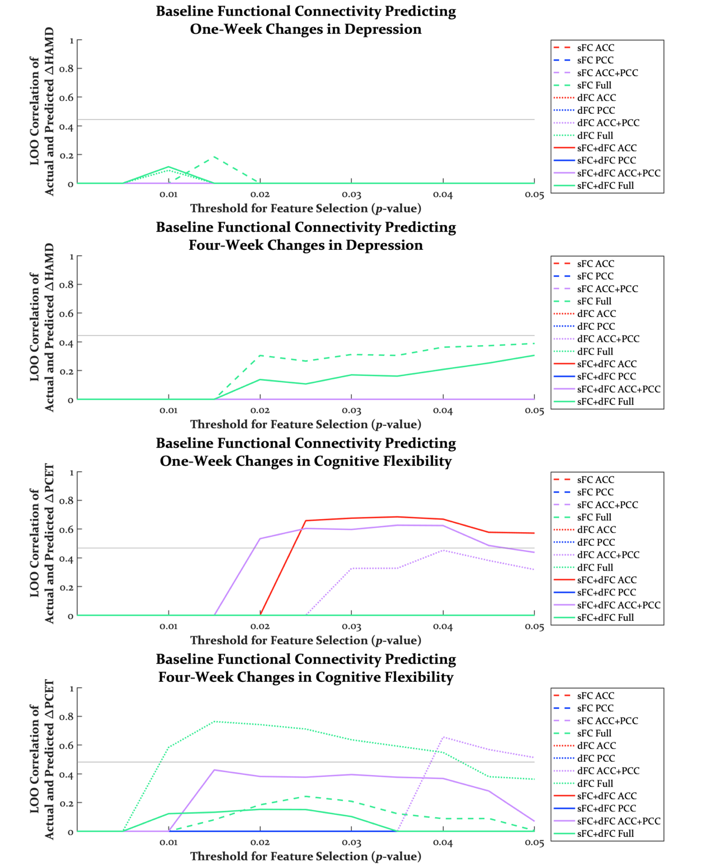


**Figure S9.** Inclusion of only ROIs with acceptable signal-to-noise in 100% of within-sphere voxels across all scans. Performance of models trained on baseline functional connectivity as a function of the threshold for feature selection predicting one-week **(a)** and four-week **(b)** changes in depression (ΔHAMD) and one-week **(c)** and four-week **(d)** changes in cognitive flexibility (ΔPCET). Horizontal grey lines indicate model performance at *p* = .05. LOO = leave-one-out, sFC = static functional connectivity, dFC = dynamics of functional connectivity, ACC = anterior cingulate cortex, PCC = posterior cingulate cortex.

**
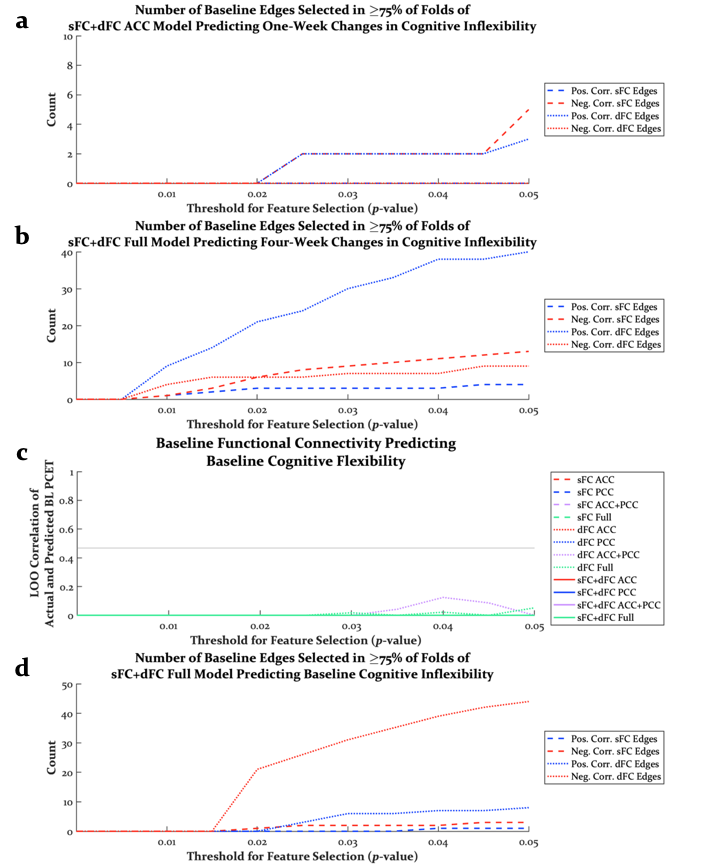
**

**Figure S10.** Inclusion of only ROIs with acceptable signal-to-noise in 100% of within-sphere voxels across all scans. Positively and negatively correlated features that were selected in the best models trained on baseline sFC and dFC predicting **(a)** one-week changes and **(b)** four-week changes in cognitive flexibility. Positively correlated dFC edges were consistently selected in these models, suggesting that greater baseline dFC was associated with more PCET perseverative errors (i.e., greater cognitive inflexibility). In contrast, when models were trained on baseline dFC to predict baseline cognitive flexibility **(c)**, dFC edges were still predictive of cognitive flexibility, but the correlations between dFC edges and cognitive flexibility was reversed **(d)**. The sFC+dFC Full model was plotted here to highlight that regardless of how many edges were allowed into the model, far more dFC edges were negatively correlated with PCET perseverative errors. Pos. = positively, Neg. = negatively, Corr. = correlated, LOO = leave-one-out, sFC = static functional connectivity, dFC = dynamics of functional connectivity, ACC = anterior cingulate cortex, PCC = posterior cingulate cortex.

**
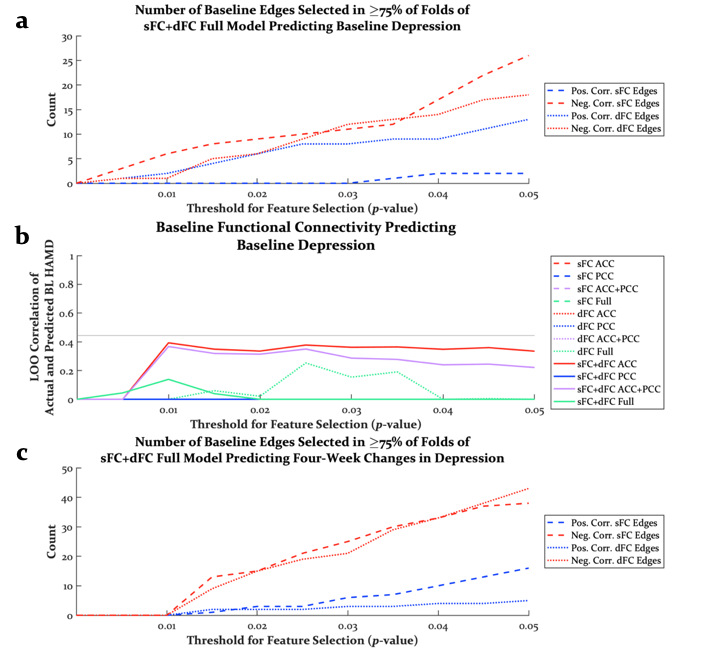
**

**Figure S11.** Inclusion of only ROIs with acceptable signal-to-noise in 100% of within-sphere voxels across all scans. The number of baseline sFC and dFC edges positively and negatively correlated with four-week changes in depression. **(b)** Performance of models trained on baseline functional connectivity as a function of the threshold for feature selection predicting predicting baseline depression (HAMD). Horizontal grey line indicates model performance at *p* = .05. **(c)** The number of baseline sFC and dFC edges positively and negatively correlated with baseline depression. Pos. = positively, Neg. = negatively, Corr. = correlated, LOO = leave-one-out, sFC = static functional connectivity, dFC = dynamics of functional connectivity, ACC = anterior cingulate cortex, PCC = posterior cingulate cortex.

**Tables**

*Effects of Psilocybin Therapy on Cognition*

**Table S1.** Dependent variables from all cognitive tasks. Columns labeled with *M* represent means and standard errors in parentheses. DV = dependent variable, -8 W = 8 weeks prior to baseline measurement in the delayed treatment group, BL = baseline, 1 W = 1 week post-psilocybin therapy, 4 W = 4 weeks post-psilocybin therapy, *F*_pre-post_ = *F*-statistic from ANOVA comparing pre- and post-psilocybin measures, *p*_F_ = *p*-value from *F*-statistic, *t*_del_ = *t*-statistic comparing both pre-psilocybin measures in the delayed treatment group to account for potential practices effects, *p_t_* = *p*-value from *t*-statistic, PCET = Penn Conditional Exclusion Task, CR = correct response, RT = median response time, ER = erroneous responses, TOT = total responses, PER ER = perseverative errors, SPVRT = Short Penn Verbal Reasoning Test, Neut = neutral, HR = hit rate, IN = neutral trials preceded by incongruent trials, NI = incongruent trials preceded by neutral trials, NN = neutral trials preceded by neutral trials, II = incongruent trials preceded by incongruent trials.

| DV | *M*_-8 W_ | *M*_BL_ | *M*_1 W_ | *M*_4 W_ | *F*_pre-post_ | *p_F_* | *t_del_* | *p_t_* |
| --- | --- | --- | --- | --- | --- | --- | --- | --- |
| PCET_CR_ | 37.70 (1.60) | 40.48 (1.68) | 37.52 (1.92) | 33.95 (1.42) | 6.23 | .004 | 3.10 | .013 |
| PCET_RT CR_ | 2840.40 (491.65) | 2808.90 (407.15) | 1984.71 (132.90) | 1970.10 (132.68) | 3.59 | .037 | 1.04 | .326 |
| PCET_ER_ | 28.60 (6.61) | 26.24 (3.70) | 13.52 (2.02) | 10.57 (1.90) | 12.73 | .000 | .32 | .760 |
| PCET_ER RT_ | 4525.80 (723.87) | 4411.64 (551.24) | 3268.60 (263.64) | 2993.76 (337.78) | 3.58 | .037 | .55 | .594 |
| PCET_PER ER_ | 16.70 (4.45) | 14.05 (2.20) | 7.38 (1.39) | 5.05 (1.10) | 10.90 | .000 | .09 | .931 |
| SPVRT_CR_ | 20.56 (.90) | 22.05 (.79) | 21.90 (.60) | 22.14 (.60) | .05 | .952 | 1.88 | .097 |
| SPVRT_RT CR_ | 11119.22 (1177.58) | 9834.21 (838.28) | 9378.98 (1032.71) | 8775.45 (673.69) | .73 | .489 | .34 | .741 |
| SPVRT_RT ER_ | 20250.79 (3252.91) | 17858.70 (2286.24) | 14089.35 (2830.86) | 14251.05 (1847.04) | 1.27 | .304 | .42 | .687 |
| Stroop_Word Neut HR_ | .99 (.00) | .98 (.00) | .98 (.00) | .98 (.00) | .71 | .497 | .43 | .678 |
| Stroop_Word Neut RT_ | -6.72 (.04) | -6.77 (.04) | -6.71 (.04) | -6.69 (.04) | 4.91 | .012 | .53 | .607 |
| Stroop_Word Neu_ *_d_*_’_ | 4.45 (.12) | 4.35 (.08) | 4.43 (.10) | 4.22 (.10) | 1.83 | .174 | .58 | .573 |
| Stroop_Word Incong HR_ | .99 (.00) | .98 (.00) | .98 (.00) | .98 (.00) | .03 | .970 | 1.86 | .096 |
| Stroop_Word Incong RT_ | -6.81 (.04) | -6.86 (.04) | -6.81 (.04) | -6.79 (.04) | 5.10 | .011 | 2.26 | .051 |
| Stroop_Word Incong_ *_d_*_’_ | 4.51 (.07) | 4.29 (.11) | 4.31 (.10) | 4.28 (.11) | .02 | .981 | 1.82 | .102 |
| Stroop_Word IN HR_ | .98 (.00) | .97 (.00) | .97 (.01) | .97 (.00) | .86 | .431 | 1.03 | .329 |
| Stroop_Word IN RT_ | -6.70 (.04) | -6.78 (.04) | -6.71 (.04) | -6.69 (.04) | 6.13 | .005 | 1.06 | .317 |
| Stroop_Word IN_ *_d_*_’_ | 4.02 (.07) | 3.93 (.06) | 3.82 (.11) | 3.81 (.08) | .78 | .464 | 1.06 | .317 |
| Stroop_Word NI HR_ | .98 (.00) | .97 (.00) | .97 (.00) | .97 (.00) | .27 | .765 | 1.80 | .105 |
| Stroop_Word NI RT_ | -6.81 (.03) | -6.88 (.04) | -6.82 (.04) | -6.79 (.04) | 4.60 | .016 | 2.66 | .026 |
| Stroop_Word NI_ *_d_*_’_ | 4.01 (.08) | 3.80 (.10) | 3.87 (.08) | 3.81 (.09) | .25 | .778 | 1.80 | .105 |
| Stroop_Word NN HR_ | .97 (.01) | .97 (.00) | .98 (.00) | .97 (.00) | 3.40 | .043 | .42 | .685 |
| Stroop_Word NN RT_ | -6.72 (.04) | -6.75 (.04) | -6.70 (.04) | -6.69 (.04) | 2.77 | .075 | .09 | .932 |
| Stroop_Word NN_ *_d_*_’_ | 3.90 (.16) | 3.90 (.08) | 4.08 (.02) | 3.87 (.07) | 4.14 | .023 | .14 | .890 |
| Stroop_Word II HR_ | .98 (.00) | .97 (.00) | .97 (.00) | .97 (.00) | .11 | .898 | 1.14 | .282 |
| Stroop_Word II RT_ | -6.80 (.05) | -6.84 (.04) | -6.79 (.04) | -6.77 (.04) | 3.38 | .044 | 1.00 | .344 |
| Stroop_Word II_ *_d_*_’_ | 3.98 (.07) | 3.94 (.06) | 3.89 (.07) | 3.93 (.07) | .10 | .903 | 1.08 | .308 |
| Stroop_Word Interference_ | -0.19 (.04) | -0.18 (.04) | -0.19 (.04) | -0.18 (.04) | .03 | .972 | 1.64 | .136 |
| Stroop_Word Gratton_ | -0.09 (.03) | -0.12 (.02) | -0.11 (.02) | -0.10 (.03) | .42 | .661 | 2.50 | .034 |
| Stroop_Ink Neut HR_ | .99 (.00) | .98 (.00) | .98 (.00) | .98 (.00) | .09 | .915 | 1.77 | .111 |
| Stroop_Ink Neut RT_ | -6.69 (.04) | -6.69 (.05) | -6.69 (.04) | -6.66 (.04) | 2.13 | .132 | -0.15 | .881 |
| Stroop_Ink Neu_ *_d_*_’_ | 4.62 (.00) | 4.40 (.11) | 4.30 (.10) | 4.38 (.10) | .35 | .703 | 1.85 | .098 |
| Stroop_Ink Incong HR_ | .98 (.01) | .98 (.00) | .98 (.00) | .98 (.00) | 1.22 | .306 | .54 | .601 |
| Stroop_Ink Incong RT_ | -6.82 (.06) | -6.84 (.05) | -6.80 (.04) | -6.82 (.05) | 1.02 | .370 | .73 | .482 |
| Stroop_Ink Incong_ *_d_*_’_ | 4.27 (.21) | 4.08 (.10) | 4.29 (.09) | 4.30 (.12) | 2.27 | .116 | 1.15 | .281 |
| Stroop_Ink IN HR_ | .98 (.00) | .97 (.01) | .97 (.00) | .98 (.00) | 1.11 | .341 | 1.31 | .222 |
| Stroop_Ink IN RT_ | -6.71 (.05) | -6.69 (.05) | -6.69 (.04) | -6.66 (.04) | 1.33 | .275 | .72 | .490 |
| Stroop_Ink IN_ *_d_*_’_ | 4.08 (.03) | 3.91 (.11) | 3.95 (.06) | 4.03 (.04) | .68 | .510 | 1.27 | .236 |
| Stroop_Ink NI HR_ | .97 (.01) | .97 (.00) | .97 (.00) | .97 (.00) | .26 | .769 | .25 | .805 |
| Stroop_Ink NI RT_ | -6.82 (.05) | -6.84 (.05) | -6.80 (.04) | -6.81 (.04) | 1.12 | .335 | 1.06 | .319 |
| Stroop_Ink NI_ *_d_*_’_ | 3.91 (.19) | 3.81 (.10) | 3.89 (.08) | 3.84 (.09) | .20 | .817 | .54 | .600 |
| Stroop_Ink NN HR_ | .98 (.00) | .98 (.00) | .97 (.00) | .97 (.00) | 1.31 | .282 | 1.55 | .154 |
| Stroop_Ink NN RT_ | -6.67 (.04) | -6.68 (.05) | -6.69 (.04) | -6.66 (.04) | 1.36 | .269 | .30 | .771 |
| Stroop_Ink NN_ *_d_*_’_ | 4.03 (.04) | 3.94 (.04) | 3.81 (.09) | 3.81 (.10) | .99 | .381 | 1.54 | .157 |
| Stroop_Ink II HR_ | .97 (.01) | .96 (.00) | .97 (.00) | .97 (.00) | 1.10 | .344 | 1.21 | .256 |
| Stroop_Ink II RT_ | -6.82 (.08) | -6.83 (.05) | -6.80 (.04) | -6.81 (.05) | .53 | .593 | .35 | .733 |
| Stroop_Ink II_ *_d_*_’_ | 3.77 (.13) | 3.70 (.09) | 3.85 (.06) | 3.88 (.09) | 1.43 | .251 | .97 | .360 |
| Stroop_Ink Interference_ | -0.15 (.03) | -0.16 (.03) | -0.11 (.03) | -0.15 (.02) | 1.41 | .257 | 1.64 | .136 |
| Stroop_Ink Gratton_ | -0.26 (.07) | -0.30 (.04) | -0.22 (.04) | -0.30 (.04) | 2.66 | .082 | 1.32 | .218 |

**Table S2.** Concentration and quality parameters of glutamate and *N*-acetylaspartate (NAA) as reported from magnetic resonance spectroscopy. Linewidth of NAA in Hz was calculated using FID-A ^7^. Signal of total creatine (tCr) was used as internal referencing standard with tCr = 8 mmol/L. L Hipp = left hippocampus, R Hipp = right hippocampus, ACC = anterior cingulate cortex, CRLB = Cramer-Rao Lower Bound, SNR = signal to noise ratio as reported in LCModel.

|  |  | Pre-Psilocybin | | Post-Psilocybin | |
| --- | --- | --- | --- | --- | --- |
|  |  | Glutamate | NAA | Glutamate | NAA |
| ACC | Concentration [mmol/l] | 10.54 ± 1.14 | 9.95 ± 0.89 | 9.75 ± 1.10 | 9.69 ± 0.66 |
|  | CRLB | 2.23 ± 0.40 | 1.68 ± 0.73 | 2.59 ± 2.42 | 1.82 ± 1.12 |
|  | SNR | 36.68 ± 10.53 | | 38.73 ± 10.10 | |
|  | Linewidth [NAA,Hz] | 21.03 ± 17.63 | | 17.89 ± 15.34 | |
| L Hipp | Concentration [mmol/l] | 8.13 ± 1.03 | 8.26 ± 1.66 | 7.61 ± 1.47 | 7.73 ± 1.62 |
|  | CRLB | 5.79 ± 1.65 | 4.74 ± 1.91 | 9.37 ± 17.65 | 4.67 ± 2.06 |
|  | SNR | 10.42 ± 3.34 | | 10.47 ± 3.44 | |
|  | Linewidth [NAA,Hz] | 38.71 ± 17.86 | | 39.13 ± 18.73 | |
| R Hipp | Concentration [mmol/l] | 7.41 ± 1.55 | 7.51 ± 2.26 | 7.19 ± 1.35 | 8.05 ± 1.61 |
|  | CRLB | 5.05 ± 1.68 | 5.26 ± 6.23 | 5.00 ± 1.15 | 3.63 ± 1.34 |
|  | SNR | 13.00 ± 3.04 | | 12.58 ± 2.81 | |
|  | Linewidth [NAA,Hz] | 38.33 ± 13.60 | | 36.03 ± 13.82 | |

**References**

1 Kurtz M. The Penn Conditional Exclusion Test: a new measure of executive-function with alternate forms for repeat administration. *Archives of Clinical Neuropsychology* 2004; **19**: 191–201.

2 Stroop JR. Studies of interference in serial verbal reactions. *Journal of Experimental Psychology* 1935; **18**: 643–662.

3 Gratton G, Coles MGH, Donchin E. Optimizing the use of information: Strategic control of activation of responses. *Journal of Experimental Psychology: General* 1992; **121**: 480–506.

4 Bilker WB, Wierzbicki MR, Brensinger CM, Gur RE, Gur RC. Development of Abbreviated Eight-Item Form of the Penn Verbal Reasoning Test. *Assessment* 2014; **21**: 669–678.

5 Makowski C, Lepage M, Evans AC. Head motion: the dirty little secret of neuroimaging in psychiatry. *Journal of Psychiatry & Neuroscience* 2019; **44**: 62–68.

6 Satterthwaite TD, Wolf DH, Loughead J, Ruparel K, Elliott MA, Hakonarson H *et al.* Impact of in-scanner head motion on multiple measures of functional connectivity: Relevance for studies of neurodevelopment in youth. *NeuroImage* 2012; **60**: 623–632.

7 Simpson R, Devenyi GA, Jezzard P, Hennessy TJ, Near J. Advanced processing and simulation of MRS data using the FID appliance ( FID‐A )—An open source, MATLAB ‐based toolkit. *Magn Reson Med* 2017; **77**: 23–33.
